# Rethinking microbial symbioses

**DOI:** 10.1093/femsle/fnz255

**Published:** 2020-03-20

**Authors:** Daniel Tamarit, Siv G E Andersson

**Affiliations:** Molecular Evolution, Science for Life Laboratory, Uppsala University, Husargatan 3, 752 37, Uppsala, Sweden

When Anton de Bary famously coined the term *symbiosis* to allude to ‘dissimilarly named organisms living together’, he was convinced that the importance and ubiquity of symbioses had already been observed:

*Was ich vorbrachte, enthält keine einzige neue Beobachtung, es sind lauter bekannte Dinge. Die Belege für die Fundamentalsätze derLehre, von der die Rede war, begegnen uns in der That, nachdem das Ei des Columbus einmal aufrecht dasteht, allerorten. Manbraucht sich nur aufmerksam umzusehen*.

*de Bary (1879)*

*What I put forward does not contain a single new observation, they are all known things. The evidence of the fundamental principlesof this doctrine, we encounter, in fact, once the egg of Columbus stands upright, everywhere. One only has to look carefully*.

*Translated from de Bary (1879)*

Indeed, de Bary focusses his treatise on describing known cases that populate the parasite–commensalist–mutualist continuum, such as lichens, cyanobacterium–plant symbioses, and multiple animal and plant pathogens. While the various examples he presented were public knowledge, de Bary's celebrated contribution prominently resides on the synthesis, evaluation and popularization of the generality of symbiotic associations. This being a paradigmatic example, the ever so multidisciplinary and composite research field of microbial symbiosis is powered by a rich tradition of reflection over the discipline's development.

Microbial symbioses are the target of a relentless army of investigators unraveling the connections between hosts and their microbes, or between *dissimilarly named* microbes. Year after year, the application of top-of-the-line methodology fuels our understanding of microbial symbioses, and the bottomless list of inspected symbiotic systems grows inexorably. The present thematic series was created to serve as a platform for contemplation and proposal. All in all, a total of 15 contributions have faced central topics in the field of microbial symbioses.

Several of these publications address major concerns in broad aspects around microbial symbioses, and come to synthetic conclusions or indicate promising avenues for future research. For example, the principles behind the evolutionary origins of mutualism are poorly understood and have been subject to debate, with various authors suggesting that mutualistic relationships may originate from parasitic interactions. In this series, Sorensen *et al*. (2019) discuss theoretical and experimental evidence supporting exploitation strategies as precursory states of mutualisms. Under this model, the host captures the microbial symbiont and the eventual evolution of mutualism depends on fitness trade-offs between symbiotic and free-living states.

Extracting general patterns from the vast amount of existing data is a necessary challenge. Much of the microbial symbioses literature is devoted to the description and analysis of specific symbiotic systems, with certain principles arising clearly, while others remain murky. The modes of transmission of microbial symbionts play an important role in infection dynamics and have major implications to host effects; consequently, understanding general factors governing transmission dynamics is hugely desirable. Russell (2019) correlates the transmission mode of characterized microbial symbionts to the environment of their hosts, finding that vertical and horizontal transmissions are more common on land and aquatic symbioses, respectively, and bringing forward the widespread nature of mixed transmission modes. Yet, a hurdle to adequately integrate our knowledge of symbiotic systems with heterogeneous properties is the lack of a formal framework to describe, compare and classify symbioses. In another publication in this series, Pacheco and Segre (2019) examine a diverse set of characterized symbiotic interactions in order to undertake such a conceptual classification. Their initial, expandable framework is based on nine qualitative features (such as dependence on physical contact, ecological outcome or habitat), and they showcase how it can aid across-system comparisons.

A strong emphasis in microbial symbioses research is put on elaborating mechanistic models for host–symbiont interactions at the molecular scale. Two publications in this series aim to decipher the molecular language used by symbiotic partners. Frank (2019) reviews eukaryotic-like proteins encoded by the prokaryotic members of microbiomes from diverse host taxonomic groups. Thairu and Hansen (2019), on the other hand, address the emerging role of small RNAs as part of symbiotic molecular languages. A common feature of bacterial endosymbionts is an overproduction of molecular chaperones. In this series, Aguilar-Rodriguez, Fares and Wagner (2019) experimentally evolved *Escherichia coli* strains to investigate role of chaperones in mitigating mutations affecting metabolic functions, which commonly occur during genome reduction processes.

The last years have seen a rapid increase in the discovery of key connections between animal physiology and the associated microbiomes. The deepening of our understanding of animal obligatory mutualisms has walked hand in hand with the incorporation of the microbiome in renovated models of health and disease, particularly in humans. This research has generated a level of enthusiasm that has perhaps led to a default, somewhat uncritical conception of mutualistic relationships being necessary for most aspects of animal physiology. Hammer, Sanders and Fierer (2019) reflect on how and why this idea has perfused biological thinking, and defend that the reliance of animals in microbiomes exists as a continuum between strictly dependent and microbiome-free species. Taking microbiome independence as a null hypothesis is therefore a logical epistemological shift, and this publication suggests methodological approaches to address it moving forward.

## ENDLESS FORMS

Microbial symbioses convey a formidable potential to facilitate adaptation to novel niches, consequently generating biodiversity. This series includes a number of system-specific contributions that offer progress into comprehending salient issues in these systems.

The formation and development of certain symbioses are influenced by the interaction of host and multiple microbial strains, resulting in the establishment and maintenance of specific, successful symbionts. Using legume-rhizobium as a model system, Younginger and Friesen (2019) argue in favor of partner choice, where hosts discriminate compatible symbionts before infection, as a key establishing mechanism, and defend a renewed focus on the study of this process.

Insect symbioses have been a frontrunner in symbiosis research for decades, with multiple paradigmatic host–bacterial associations. Particularly, bacteria of the genus *Wolbachia* are capable of a variety of symbiotic associations with arthropods and display a colorful set of strategies with heavy bearings on host fitness. Here, Lopez-Madrigal and Duarte (2019) review factors influencing the regulation of *Wolbachia* loads, itself a key element influencing the effect of this symbiont on the host. Another classical symbiosis that retains the ability to surprise us is that of lichens. Here, Spribille *et al*. (2020) bring forward unanswered questions regarding species interactions, chemical construction of their environment and spatial organization in lichen biofilms.

Compensating a long-standing lack of exploration of the microbiology of ocean floors, and in view of the startling climate crisis underway, a recent focus has emerged in marine microbiology, particularly in relation to the microbial association to ecologically relevant hosts. Two publications in this series review the microbial influence in the healthy states of coral and seagrass physiology, respectively. Hernandez-Agreda, Leggat and Ainsworth (2019) discuss spatial-temporal dynamics of the coral microbiome and the standardization of amplicon sequencing techniques to improve its characterization. Tarquinio *et al*. (2019) describe the role of the seagrass microbiome in the nutrition, development and protection of the host. Additionally, this series includes a research article by Caputo, Nylander and Foster (2019) where the authors use imaging and phylogenetic analyses to study the evolution of symbiotic associations between diatoms and diazotrophic cyanobacteria.

Ancient transitions to symbiotic lifestyles are also featured in this thematic series. Cultivation-independent techniques have revealed shockingly divergent and diverse microbial groups that have profoundly reshaped the tree of life. This expansion of known diversity has opened new, fertile grounds for symbiosis studies. Major groups such as the bacterial Candidate Phyla Radiation lineage or DPANN archaea (group named after the originally included phyla: Diapherotrites, Parvarchaeota, Aenigmarchaeota, Nanoarchaeota and Nanohaloarchaeota) seem to display symbiotic features, and their biology is only now starting to be uncovered. Dombrowski *et al*. (2019) review the discovery of DPANN archaea, the progress in their genomic characterization and their phylogenetic position in the archaeal tree. Another alluring subject related to ancient symbioses is the study of the symbiogenetic origins of organelles, which dates back over 100 years and presents notable hurdles. Here, we may find extraordinary allies in unicellular eukaryotic lineages, where underexplored representatives are protagonists of fascinating interactions. Gavelis and Gile (2018) review the current view in the establishment of primary plastid symbioses, and discuss experimental lines using microbial eukaryotes to bring novel perspectives on how photosynthetic organelles originate. These approaches and other detailed investigations of novel microbial lineages hold tremendous promise to our understanding of the evolution of symbiotic lifestyles in deep branches of the tree of life.

Symbiosis research builds on a continuous and overwhelming production rate of primary research. New discoveries proceed in quick fashion, and bring captivating surprises that either add beautifully to the existing body of research or, even better, push us to reconsider the way we comprehend biological interactions. While much of the integration of knowledge into larger ecological and evolutionary frameworks takes place organically, we welcome opportunities such as the one allowed by this thematic series to assimilate and ponder about recent findings. We eagerly look forward to the next advances and the discussions they will stimulate.

## FUNDING

The authors are supported by the Swedish Research Council (2018–06609 to Daniel Tamarit and 2018–04135 to Siv G. E. Andersson) and the Knut and Alice Wallenberg Foundation (2017.0322 and 2018.0414 to Siv G. E. Andersson).

## References

[bib1] Aguilar-Rodriguez J , Fares MA, Wagner A. Chaperonin overproduction and metabolic erosion caused by mutation accumulation in *Escherichia coli*. FEMS Microbiol Lett. 2019;366:fnz121. 31150542 10.1093/femsle/fnz121

[bib3] de Bary A . Die erscheinung der symbiose. Strasbourg: Verlag Karl J Trübner, 1879.

[bib2] Caputo A , Nylander JAA, Foster RA. The genetic diversity and evolution of diatom–diazotroph associations highlights traits favoring symbiont integration. FEMS Microbiol Lett. 2019;366:fny297. 10.1093/femsle/fny297 PMC6341774 30629176

[bib4] Dombrowski N , Lee JH, Williams TA et al. Genomic diversity, lifestyles and evolutionary origins of DPANN archaea. FEMS Microbiol Lett. 2019;366:fnz008. 10.1093/femsle/fnz008 PMC6349945 30629179

[bib5] Frank AC . Molecular host mimicry and manipulation in bacterial symbionts. FEMS Microbiol Lett. 2019;366:fnz038. 30877310 10.1093/femsle/fnz038

[bib6] Gavelis GS , Gile GH. How did cyanobacteria first embark on the path to becoming plastids?: lessons from protist symbioses. FEMS Microbiol Lett. 2018;365:fny209. 10.1093/femsle/fny209 30165400

[bib7] Hammer TJ , Sanders JG, Fierer N. Not all animals need a microbiome. FEMS Microbiol Lett. 2019;366:fnz117. 31132110 10.1093/femsle/fnz117

[bib8] Hernandez-Agreda A , Leggat W, Ainsworth TD. A place for taxonomic profiling in the study of the coral prokaryotic microbiome. FEMS Microbiol Lett. 2019;366:fnz063. 30939203 10.1093/femsle/fnz063

[bib9] Lopez-Madrigal S , Duarte EH. Titer regulation in arthropod–Wolbachia symbioses. FEMS Microbiol Lett. 2019:fnz232. 31750894 10.1093/femsle/fnz232

[bib10] Pacheco AR , Segre D. A multidimensional perspective on microbial interactions. FEMS Microbiol Lett. 2019;366:fnz125. 31187139 10.1093/femsle/fnz125 PMC6610204

[bib11] Russell SL . Transmission mode is associated with environment type and taxa across bacteria–eukaryote symbioses: a systematic review and meta-analysis. FEMS Microbiol Lett. 2019;366:fnz013. 30649338 10.1093/femsle/fnz013

[bib12] Sorensen MES , Lowe CD, Minter EJA et al. The role of exploitation in the establishment of mutualistic microbial symbioses. FEMS Microbiol Lett. 2019;366:fnz148. 31271421 10.1093/femsle/fnz148 PMC6638607

[bib1_330_1583753664496] Spribille T , Tagirdzhanova G, Goyette S et al. 3D biofilms: in search of the polysaccharides holding together lichen symbioses. FEMS Microbiol Lett. 2020;; fnaa023. 32037451 10.1093/femsle/fnaa023 PMC7164778

[bib14] Tarquinio F , Hyndes GA, Laverock B et al. The seagrass holobiont: understanding seagrass–bacteria interactions and their role in seagrass ecosystem functioning. FEMS Microbiol Lett. 2019;366:fnz057. 30883643 10.1093/femsle/fnz057

[bib15] Thairu MW , Hansen AK. It's a small, small world: unravelling the role and evolution of small RNAs in organelle and endosymbiont genomes. FEMS Microbiol Lett. 2019;366:fnz049. 30844054 10.1093/femsle/fnz049

[bib16] Younginger BS , Friesen ML. Connecting signals and benefits through partner choice in plant–microbe interactions. FEMS Microbiol Lett. 2019;366:fnz217. 31730203 10.1093/femsle/fnz217

